# Analyzing the impact of trade and investment agreements on pharmaceutical policy: provisions, pathways and potential impacts

**DOI:** 10.1186/s12992-019-0518-2

**Published:** 2019-11-28

**Authors:** Deborah Gleeson, Joel Lexchin, Ronald Labonté, Belinda Townsend, Marc-André Gagnon, Jillian Kohler, Lisa Forman, Kenneth C. Shadlen

**Affiliations:** 1 School of Psychology and Public Health, La Trobe University, Melbourne, Australia; 2 School of Health Policy & Management, York University, Toronto, Canada; 3 School of Epidemiology and Public Health, University of Ottawa, Ottawa, Canada; 4 School of Regulation and Global Governance, Australian National University, Canberra, Australia; 5 School of Public Policy & Administration, Carleton University, Ottawa, Canada; 6 Leslie Dan Faculty of Pharmacy, University of Toronto, Toronto, Canada; 7 Dalla Lana School of Public Health, University of Toronto, Toronto, Canada; 8 Department of International Development, London School of Economics and Political Science, London, UK

**Keywords:** Trade agreements, Access to medicines, Pharmaceutical policy, Pharmaceuticals, Trans-Pacific Partnership, Comprehensive and Progressive Agreement on Trans-Pacific Partnership, Comprehensive Economic and Trade Agreement, CPTPP, TPP-11, United States-Mexico-Canada Agreement, TRIPS

## Abstract

**Background:**

Trade and investment agreements negotiated after the World Trade Organization’s Agreement on Trade-Related Aspects of Intellectual Property Rights (TRIPS) have included increasingly elevated protection of intellectual property rights along with an expanding array of rules impacting many aspects of pharmaceutical policy. Despite the large body of literature on intellectual property and access to affordable medicines, the ways in which other provisions in trade agreements can affect pharmaceutical policy and, in turn, access to medicines have been little studied. There is a need for an analytical framework covering the full range of provisions, pathways, and potential impacts, on which to base future health and human rights impact assessment and research. A framework exploring the ways in which trade and investment agreements may affect pharmaceutical policy was developed, based on an analysis of four recently negotiated regional trade agreements. First a set of core pharmaceutical policy objectives based on international consensus was identified. A systematic comparative analysis of the publicly available legal texts of the four agreements was undertaken, and the potential impacts of the provisions in these agreements on the core pharmaceutical policy objectives were traced through an analysis of possible pathways.

**Results:**

An analytical framework is presented, linking ten types of provisions in the four trade agreements to potential impacts on four core pharmaceutical policy objectives (access and affordability; safety, efficacy, and quality; rational use of medicines; and local production capacity and health security) via various pathways.

**Conclusions:**

The analytical framework highlights provisions in trade and investment agreements that need to be examined, pathways that should be explored, and potential impacts that should be taken into consideration with respect to pharmaceutical policy. This may serve as a useful checklist or template for health and human rights impact assessments and research on the implications of trade agreements for pharmaceuticals.

## Background

Over two decades ago, the 1995 World Trade Organization’s Agreement on Trade-Related Aspects of Intellectual Property Rights (the TRIPS Agreement) set minimum global standards for intellectual property rights that included committing members to providing patent terms of at least 20 years for pharmaceuticals (initiated from the date of filing) [1, 2].^1^ Since this time, subsequent bilateral and regional trade agreements, particularly those negotiated by the USA and the EU (where most of the global research-based pharmaceutical industry is headquartered), have progressively expanded and extended intellectual property (IP) protection beyond the requirements of TRIPS through a multitude of additional provisions: the “TRIPS-Plus” protections [1–3].

There is a large body of literature analyzing the potential negative effects of intellectual property rules incorporated in various trade agreements on access to medicines, and how these rules operate (see, for example, [1, 4–6]). A handful of empirical studies have debated the effects on the timing of generic market entry, medicine prices or expenditure, or access to medicines (see, for example, [7–11]).

Trade agreements also include provisions, beyond IP, that can impact on pharmaceutical policy and practice. For example, US trade agreements with Australia and Korea have included provisions applying to national pharmaceutical coverage programs and regulation of pharmaceutical marketing [3], and similar rules subsequently appeared in the Trans-Pacific Partnership Agreement (TPP) [12] and the United States-Mexico-Canada Agreement (USMCA) [13]. The TPP also included a set of novel provisions focused on the assessment of safety and efficacy [14], which were subsequently incorporated in the USMCA [13]. These provisions have been less extensively explored. Given the sheer scope and breadth of the legal rules negotiated in recent trade agreements, there is an increasing number of potential intersections between trade and investment rules and pharmaceutical policy, going beyond the familiar territory of IP and access to medicines, with a range of implications for UN Sustainable Development Goal (SDG) 3.8 (“Achieve universal health coverage, including financial risk protection, access to quality essential health-care services and access to safe, effective, quality and affordable essential medicines and vaccines for all”) [15]. Many provisions now commonly included in trade agreements can impinge on access to safe, effective, quality and affordable medicines, potentially undermining the achievement of universal health coverage and the SDGs.

Thus, a more comprehensive understanding of the potential points of intersection and impacts of trade agreements on national pharmaceutical policy is needed, to inform health and human rights impact assessments^2^ of trade agreements under negotiation (to the extent that negotiating texts are made public or leaked) or to be undertaken in the future, and research into their effects following implementation. This paper aims to help fill this gap by identifying the provisions in recently negotiated regional trade and investment agreements that are relevant to pharmaceutical policy and practice, tracing the pathways through which they can affect pharmaceutical policy objectives (how they may interact with pharmaceutical policy to produce impacts on specific pharmaceutical policy objectives), and developing a framework for analyzing the impact of trade and investment agreements on pharmaceutical policy and access to medicines.

## Methods

The first step in developing the analytical framework involved identifying a set of core pharmaceutical policy objectives on which there is international consensus, or which have been commonly adopted in pharmaceutical policy: (i) access and affordability; (ii) safety, efficacy, and quality; (iii) rational use of medicines, and (iv) local production capacity and health security. These objectives, initially identified by the first author and then discussed and agreed among all authors, were based on SDG 3.8 [15] and on the WHO advice to member states on how to develop and implement a pharmaceutical policy [17]. SDG 3.8 emphasizes the need for “access to safe, effective, quality and affordable medicines” in order to achieve universal health coverage [15]. The WHO advice on “general objectives of a national drug policy” includes access (understood as “equitable availability and affordability of essential drugs”); quality, safety, and efficacy; and rational use of medicines [17]. Strengthening “national pharmaceutical production capacity” is also recognized as a legitimate national drug policy goal for some countries [17]. It is important to note that there can be tensions between these policy objectives that require careful negotiation in a nation’s pharmaceutical policy: for example, affordability must be balanced with the need to ensure safety and efficacy, and local production must be carefully balanced with affordability. However, a 2011 WHO report on local production concluded that local production is one means by which governments in developing countries can maintain a balance between the availability of quality products and meeting priority public health needs with products that are acceptable and affordable [18].

Next, we undertook a systematic, comparative analysis of the legal texts of four recently concluded trade and investment agreements in order to identify a comprehensive set of provisions relevant to pharmaceuticals. The agreements selected were:

- the Trans-Pacific Partnership Agreement (TPP) [12], negotiated among twelve countries and signed in February 2016, but stalled since the US withdrawal in January 2017.
- the Comprehensive and Progressive Agreement for Trans-Pacific Partnership (CPTPP or TPP-11) [19]; the agreement that was salvaged from the TPP by the remaining 11 countries. This incorporates most of the legal text from the TPP, but with certain provisions suspended. It was signed in March 2018 and came into force for the first six countries to complete their domestic approval processes (Australia, Canada, Japan, Mexico, New Zealand, and Singapore) in December 2018, and for Vietnam in January 2019.^3^
- the Comprehensive Economic and Trade Agreement (CETA) [20] between the EU and Canada, which was signed in October 2016 and provisionally entered into force in September 2017.
- the US-Canada-Mexico Agreement (USMCA) [13], signed in November 2018 but not yet in force at the time of writing.

These four agreements were selected because they are large regional trade agreements for which negotiations concluded within the past 3 years and for which final legal texts are publicly available. Together they represent the likely direction of binding rules affecting pharmaceutical policy in future agreements.

The publicly available legal texts of the agreements, including annexes and side instruments,^4^ were sourced from government websites (primarily the New Zealand Ministry of Foreign Affairs and Trade, Global Affairs Canada, and the Office of the United States Trade Representative).^5^ The contents of all text potentially relevant to pharmaceuticals was scanned to identify relevant provisions; next, relevant chapters, annexes, and side instruments were selected for closer study. The selection process was undertaken independently by the lead and second author, and discrepancies resolved through discussion. Provisions with potential implications for pharmaceuticals were organized into categories according to how they affect pharmaceutical policy; then mapped across the agreements and important differences noted.^6^ Relevant chapters, annexes, and side instruments in each category were analyzed independently by the lead author and another member of the research team, and discrepancies resolved through discussion. For the purpose of constructing the analytical framework, comparison of the texts of the four agreements focused on identifying provisions that could have a novel or incremental impact on pharmaceutical policy as compared to the other agreements. Major reversals in trends (such as suspension of certain provisions in the CPTPP) were also noted.

The final step involved tracing the potential impact of the provisions identified in the agreements on the core pharmaceutical policy objectives (as noted above) through an analysis of possible pathways, drawing on the authors’ disciplinary expertise and research experience in the field of trade agreements and pharmaceutical policy, and on published research evidence (where available and relevant). The aim here was to identify possible pathways and potential impacts that should be the focus of health and human rights impact assessments and scholarly research.

## Results

We identified ten types of provisions in our data set of trade agreements that could impact on domestic pharmaceutical policy and regulation:

1. TRIPS-Plus intellectual property protections;
2. Investment protections, including investor-state dispute settlement;
3. Procedural requirements for pharmaceutical pricing and reimbursement programs;
4. Provisions with implications for regulation of pharmaceutical marketing;
5. Regulatory requirements for assessment of safety, efficacy, and quality;
6. Reduction/elimination of tariffs on medicines or their ingredients;
7. Rules applying to government procurement of pharmaceuticals;
8. Rules applying to state-owned enterprises and designated monopolies;
9. Procedural requirements for customs administration and trade facilitation; and
10. Rules applying to regulatory practices, cooperation and coherence.

The pathways through which these provisions could impact on pharmaceutical policy are summarized in Table 1 and explained in each of the sections below. Additional file 1 provides a detailed breakdown of the chapters, annexes, and side instruments in which relevant provisions were found, organized by the ten types of provisions.

**Table 1:** Summary of analytic framework linking provisions, pathways, and potential impacts

Provisions	Pathways	Potential impacts on core pharmaceutical policy objectives
TRIPS-Plus intellectual property protection	• Extended periods of exclusivity for patented medicines and obstacles to market entry for generic and biosimilar medicines can reduce competition and lead governments and consumers to pay monopoly prices for longer periods of time	• Access to affordable medicines may be reduced
Investment protection: investor-state dispute settlement mechanism; investment chapter with IP covered in definition of investment	• Disputes, or the threat of a dispute, may cause reversal of pharmaceutical policy decisions or regulatory chill—possibly resulting in extended exclusivity periods, relaxation of regulatory standards or inability to support local producers	• Access to affordable medicines may be reduced • Rational use of medicines may be compromised • Local production and health security may be compromised
Procedural requirements for national pharmaceutical pricing and reimbursement programs	• Industry objectives and values may be given priority over public health and access to medicines • Pharmaceutical companies may be given additional opportunities to provide input to, or to contest, decision-making regarding pricing and/or reimbursement • Flexibility regarding prioritization and timing of listing drugs for reimbursement may decrease • Scarce health resources may be diverted towards implementing procedural requirements with no public benefit • Pharmaceutical policy-making may come under pressure from trade partners with large pharmaceutical industries • Excessive prices may not reflect clinical value of medicines	• Access to affordable medicines may be reduced • Rational use of medicines may be compromised
Provisions with implications for regulation of pharmaceutical marketing	• Attempts to prohibit or restrict pharmaceutical promotion to health professionals (to encourage better prescribing) or consumers (to encourage better use of medicines) may be reversed or chilled • Restrictions on pharmaceutical marketing may be difficult to enforce (for cross-border advertising services)	• Rational use of medicines may be compromised
Regulatory requirements for assessing safety, efficacy and quality	• Standards may be lowered through harmonization to the lowest common denominator, pressure from trade partners to adopt lower standards or greater involvement of the pharmaceutical industry in standard-setting • Pressure to speed up regulatory approval processes may result in increase in safety risks • Constraints on public information about pharmaceutical inspections may compromise safety and quality • Cooperation on pharmaceutical inspection issues may improve the quality of medicines thereby improving consumer safety	• Safety, efficacy and quality of medicines may be compromised • Manufacturing quality of medicines may be lowered or improved
Reduction/elimination of tariffs on pharmaceuticals or their ingredients	• Prices of imported pharmaceuticals may fall, in some circumstances (if additional mark-ups are not applied at other points in the supply chain) • Viability of local generic pharmaceutical industry in question if there is greater competition—potentially reducing supply and compromising health security	• Access to affordable medicines may increase • Local production and health security may be compromised
Rules applying to government procurement of pharmaceuticals	• Governments/hospitals may pay lower prices as a result of open tendering, depending on the nature of the procurement process and institutions • Viability of fledgling domestic pharmaceutical industries may be reduced if government and hospital purchasing cannot preference local suppliers	• Access to affordable medicines may increase • Local production and health security may be compromised
Rules applying to state-owned enterprises and designated monopolies	• Viability of domestic pharmaceutical industry in developing countries may be affected if state-owned pharmaceutical companies are required to operate as commercial entities and cannot be given financial support or preferential treatment, or cannot give preference to local suppliers • Pressure for reform of state owned enterprises may result in greater competition and lower prices	• Local production and health security may be compromised, or improved
Procedural requirements for customs administration and trade facilitation	• Movement of generic pharmaceuticals across borders may be impeded, or facilitated, in cases of suspected breaches of IP laws	• Access to affordable medicines may be reduced, or improved
Rules applying to regulatory practices, cooperation and coherence	• Pharmaceutical industry may have additional levers to provide input to, or contest pharmaceutical policy • Potential for industry representation on or input to expert advisory groups may compromise optimal pharmaceutical policy outcomes	• Access to affordable medicines may be reduced • Safety, efficacy and quality of medicines may be compromised • Rational use of medicines may be compromised • Local production and health security may be compromised

### TRIPS-plus intellectual property (IP) protections

Each of the four agreements (TPP, CPTPP, CETA, USMCA) contains IP chapters that include TRIPS-Plus IP provisions which, depending on a country’s existing IP laws and pharmaceutical policies, could delay generic competition and potentially impact negatively on access to medicines.^7^ For example, each of these agreements includes patent term adjustments and data protection for new pharmaceutical products. The types of TRIPS-Plus provisions common in these agreements and the ways in which they serve to prolong exclusivity and delay generic competition are summarized in Table 2 below. Additional file 2 provides the article/section numbers for the relevant IP provisions found in each agreement.

**Table 2:** Types of TRIPS-Plus IP provisions common in recent regional trade agreements

Type of provision	Mechanism for prolonging exclusivity
Requirement to grant patents for new uses of known products, new methods of using known products, or new processes of using known products	Enables firms to obtain additional patent protection for new forms or uses of existing products, which may reduce the use of equivalent versions after the expiry of primary patents on original molecules.
Patent term adjustments to compensate for delays in granting patents and/or in marketing approval processes	Extends the length of patent terms.
Data protection for new pharmaceutical products including biologics (an alternative pathway for maintaining monopoly control based on the clinical trial data submitted to regulators in order to gain marketing approval)	Can add to the length of exclusivity if the period of data protection extends beyond the expiration of relevant patents. Can provide monopoly protection for drugs or biologics that are not protected by patents (e.g., where a drug or biologic is not eligible for a patent or where the key patent has been invalidated). Provides a time-limited but absolute monopoly which cannot be challenged in court (as in the case of a patent) and may prevent or delay marketing approval of generics produced under a compulsory or government use license.
Additional data protection for new indications/formulations/methods of administration or for combination products containing a chemical entity that has not previously been approved	
New and/or longer periods of data protection or market exclusivity for biologic products;^a^	
Patent linkage mechanisms (linking patent status with marketing approval of generics);	Can extend periods of exclusivity if marketing approval is denied due to patents of questionable validity, patents that are not being infringed by the generic product or patents for changes that have no direct therapeutic applications.
Trade secrets protection	Unlike a patent, trade secret protection does not provide a time-limited monopoly, so it can potentially exclude competition indefinitely.^b^
TRIPS-Plus provisions for the enforcement of intellectual property rights	Strict enforcement of, and penalties for, suspected violations of intellectual property rights, including seizure of suspected counterfeit goods at the border (i.e., goods suspected of violating IP rules rather than being of deliberately inferior quality), excessive damages, provisional measures, and criminal enforcement of patent infringement.

^a^Biologic products are a new class of medicines which are derived from living cells using biotechnological processes and that need to be delivered by injection or intravenously. These include many expensive treatments for cancer and autoimmune diseases, and account for a growing share of the global pharmaceutical market and of pharmaceutical expenditure in many countries. [21]. IMS Institute for Healthcare Informatics. The Global Use of Medicines: Outlook through 2017. IMS Health, 2013

^b^Protection of trade secrets is likely to play an increasing role in excluding competition due to the growing dominance of biologics and the emergence of personalized medicine. The manufacturing processes for developing these newer treatments are very complex, and may make it essentially impossible to create a biosimilar that is identical to the reference product. [22]. Lyman GH, Zon R, Harvey RD, Schilsky RL. Rationale, opportunities, and reality of biosimilar medications. New England Journal of Medicine 2018;378:2036–2044

Agreements have varied as to the patterns of TRIPS-Plus IP provisions they include. The TPP included each of the TRIPS-Plus provisions indicated in Table 2 [23], though many of these were suspended in the CPTPP following the US withdrawal [24], including the requirement to provide patents for new uses, methods and processes of using existing products, and the provisions providing for patent term adjustments and data/market protection (see Additional file 2 for details). Patent linkage, trade secrets protection, and TRIPS-Plus enforcement provisions, however, were retained in the revived agreement.

The USMCA IP chapter is closely based on the corresponding chapter of the TPP, but includes 10 years of “effective market protection” for biologics, longer than the period negotiated in the TPP [25]. For Canada, this will increase the period of market protection for biologics by 2 years; two studies of the potential impact on pharmaceutical expenditure (using different methods and based on different assumptions) have estimated the savings foregone at between CDN$0 and $305.8 [26] and up to CDN$169 by 2029 [27]. The USMCA also includes a broader definition of biologics, potentially expanding the array of drugs which will be eligible for this longer period of exclusivity [25].

Overall, CETA contains fewer TRIPS-Plus provisions than the TPP or USMCA, but it provides for a longer data/market protection period for new pharmaceutical products than the TPP (although CETA did not extend data protection in Canada). CETA does not contain special data protection or market exclusivity rules for biologics, but the length of the data protection period provided for all drugs under CETA is equivalent to the length for biologics under the TPP in any case. CETA does not include a provision for patent linkage, since patent linkage is prohibited in the EU; however CETA requires Parties that rely on patent linkage mechanisms to provide the right of appeal to all litigants—which effectively enables originator manufacturers in Canada to slow down generic entry through patent litigation [28].

The IP provisions in these agreements may delay the market entry of less expensive generic and biosimilar medicines, keeping prices high for longer periods, in turn impacting on government expenditure on pharmaceuticals and/or out-of-pocket costs for consumers, depending on the health system in each country [1–8, 29].^8^ They can also “lock in” high levels of intellectual property protection, preventing or constraining reform, as revising trade agreements typically requires the consent of all Parties. Whether specific provisions in specific agreements will have these effects depends on many factors, including the existing domestic intellectual property laws in member countries, the details of their health and pharmaceutical systems and markets, and decisions of dispute resolution panels, should there be complaints.

### Investment protection

Investor-state dispute settlement (ISDS) mechanisms are included in each of the four agreements examined here. ISDS provides an avenue for foreign investors, including pharmaceutical companies, to contest the policies, decisions, and laws of governments, by bringing a claim for compensation to an international arbitral tribunal, arguing that their investor rights under the agreement have been breached. Notably, after a three-year window (during which any claims filed will proceed under the old NAFTA provisions), the USMCA provides for ISDS only between the USA and Mexico; moreover, the grounds on which a claim may be brought are significantly narrowed in comparison with the other agreements and do not apply to pharmaceuticals. CETA’s ISDS provisions have been suspended until/unless they are approved by each EU member state. After CETA was signed, its ISDS rules were also substantially revised, calling for the creation of an “Investment Court System” with professional and independent judges (rather than temporary tribunals), opening up hearings to the public, and publishing documents submitted during cases.

ISDS has become highly controversial due to the rising number of cases, including several high-profile cases over environmental and public health policies [33]. One such case was a claim for hundreds of millions of dollars in compensation by the tobacco giant Philip Morris against the Government of Australia over its tobacco plain packaging laws [34].^9^ Because of this controversy, recently negotiated investment chapters (e.g., TPP Chapter 9) have included clauses aimed at reducing the likelihood of investors winning cases against legitimate, non-discriminatory health measures. Many of these clauses are yet untested and some legal scholars have expressed doubts over the degree to which such putative safeguards will assist countries to defend claims against health and environmental policies and laws [35]. In contrast, some recently concluded bilateral trade agreements have explicitly excluded public health measures and/or specific health programs (see, for example, the Peru-Australia Free Trade Agreement, chapter 8, footnote 17) [36].

An ISDS claim, or the threat of one, may deter governments from enacting health and pharmaceutical policies: an effect known as “regulatory chill.” This occurs partly due to the prohibitive costs associated with ISDS. The Australian Government spent approximately A$23 million defending the claim by Philip Morris Asia over tobacco plain packaging [37].^10^ If the investor wins, the awards may also be substantial: investors initiating ISDS claims in 2017 sought from 15 million USD to 1.5 billion USD [38]. Considerable uncertainty attends the outcome of ISDS claims, due to various procedural issues, including the ad hoc nature of decisions (arbitrators are not bound by previous tribunal decisions), the potential for conflicts of interest among arbitrators, and the lack of an appeals process [39, 40]. While recent agreements such as the TPP have improved on some aspects of the ISDS process (like the transparency of proceedings), many procedural problems remain [40].

One area of particular concern is the use of ISDS to enforce IP rights [40]. IP is included in the definition of investment in each agreement (see, for example, TPP Art 9.1). Article 9.8.5 of the TPP is aimed at excluding compulsory licenses and the “revocation, limitation or creation of intellectual property rights” from the scope of ISDS as long as such actions are consistent with the TPP IP chapter and the TRIPS Agreement [14]. But interpreting the TRIPS Agreement outside the WTO context is risky: ISDS panels often deliver narrow interpretations that may not incorporate the full intent (or provisions) of TRIPS [40].

The most significant example of ISDS in relation to pharmaceuticals is a claim launched by the US pharmaceutical company, Eli Lilly, against the Canadian Government, after Canadian courts had invalidated patents for uses of two drugs that had been found not to provide the promised benefits [41]. Eli Lilly contested not just the specific decisions in relation to those drugs, but also how Canadian courts had relied on the claims made in the patent application to assess the utility of a patent (referred to as the promise/utility doctrine) [42]. Eli Lilly was not successful in its ISDS challenge, but the Canadian Supreme Court subsequently weakened the utility requirement, reducing the amount of evidence required for successfully defending patents—a move which some commentators have attributed to ongoing pressure from the USA and the pharmaceutical industry [42].

ISDS may have “chilling” effects on health policy even when cases do not proceed to arbitration. For example, Colombia desisted from issuing a compulsory license for imatinib (Glivec/Gleevec) after Novartis filed a notice of dispute in 2016; and Ukraine de-registered a generic hepatitis C medicine after Gilead indicated that it would pursue arbitration [42].

In addition to disputes over intellectual property rights, ISDS could potentially be used to challenge or “chill” other pharmaceutical policy decisions, such as decisions not to approve particular drugs, conditions for drug reimbursement by public drug plans, rules against the promotion of off-label use, rules about safety and inspections, or policies benefiting local producers. Possible impacts include extended exclusivity periods, relaxation of regulatory standards, less rational prescribing, and reduced viability of the domestic pharmaceutical industry.

### Procedural requirements for national pharmaceutical pricing and reimbursement programs

Three of the four agreements contain provisions that may impact a country’s pharmaceutical reimbursement program. The TPP and USMCA include a near-identical set of provisions applying to national programs for listing medicines and medical devices on national formularies and setting prices for reimbursement. The provisions include a set of aspirational principles applying to systems for pharmaceuticals and medical devices, a set of procedural rules, and the requirement to provide opportunities to consult when a written request is received from another Party.^11^ The CPTPP retains the principles and the consultation requirement from the TPP but suspends its procedural rules (see Additional file 1).

The principles in TPP Annex 26-A (Art. 2) (and retained in the CPTPP) and in USMCA Chapter 29 (Art. 29.6) are not written in treaty-level language and are not enforceable through state-to-state dispute settlement. The principles may, however, serve a normative purpose by reinforcing industry values and priorities, and could conceivably be referenced by dispute panelists in ISDS rulings. While acknowledging the “importance of protecting and promoting public health,” the principles are weighted towards pharmaceutical industry objectives, using language such as “innovation associated with research and development” and “the value of pharmaceutical products”.

More significant are the procedural rules contained in both the TPP (Annex 26-A, Art. 3) and USMCA (Art. 29.7). These rules, which were suspended in the CPTPP, include requirements to: complete assessment of applications within a specified time-period, disclose “procedural rules, methodologies, principles and guidelines” used for their assessment, provide “timely” opportunities for applicants to comment during decision-making, and provide written information about the reasons for decisions. Furthermore, countries must provide a review process for negative listing decisions, which may be invoked at the request of an affected applicant.

Unless carefully managed, these rules may facilitate industry input (and potentially interference) in decision-making on listing and reimbursement of pharmaceuticals, as well as reducing flexibility in the prioritization and timing of listing decisions. In the event of a dispute brought by a pharmaceutical company using the ISDS mechanism in the TPP and CPTPP, the rules might be used to lend weight to industry arguments, for example, regarding the rights of investors to a minimum standard of treatment [14]. At the very least, compliance with the requirements entails committing resources to administering processes that serve the interests of industry, rather than useful public purposes.

The procedural rules in the TPP were suspended in the CPTPP. Had they been retained, as an example of their potential implications if introduced in future trade agreements, New Zealand would have been required to introduce a statutory timeframe for assessing applications for public funding and a review process for negative listing decisions—at an estimated cost of NZ $4.5 million initially and $2.2 million per year in ongoing costs (approx. 10% of the operating costs of New Zealand’s Pharmaceutical Management Agency) [43].

Currently neither Canada nor Mexico has national reimbursement programs that will fall under the procedural requirements in the USMCA^12^; their inclusion in this agreement perhaps anticipates a future national “Pharmacare” scheme in Canada. Such a scheme would have to abide by the legally binding rules in the USMCA, although Canada’s interpretation and implementation of these rules would not be subject to formal dispute settlement procedures.

A third set of provisions in the TPP, CPTPP, and USMCA relevant to pharmaceutical reimbursement is the requirement to “afford adequate opportunity” for consultation on receipt of a written request from another Party (TPP Annex 26-A, Art. 5 and USMCA Art. 29.9). However, health officials must be involved in these consultations—which may well make it less likely that such consultations become platforms for pharmaceutical lobbying. The terms of reference of a medicines working group established by similar provisions in the Australia-US FTA were carefully circumscribed, and initial fears about its impact on pharmaceutical policy in Australia proved groundless [3, 14]. All the same, the consultation requirement remains a risk for countries that are more vulnerable to pressure from other Parties, or in circumstances where the consultation process may not be similarly circumscribed.

It is important to note that, although the rules for pharmaceutical pricing and reimbursement programs are framed in terms of transparency, and the trade agreements studied also include other provisions of more general application, framed in terms of promoting transparency and anti-corruption, these appear to be largely window-dressing. For example, they lack operational definitions of “transparency” and “corruption,” lack effective accountability mechanisms, and largely ignore the private sector. As such, these agreements are unlikely to contribute much to promoting transparency and anti-corruption in the pharmaceutical sector, despite the importance of these issues.

### Provisions with implications for regulation of pharmaceutical marketing

Three of the four agreements contain specific provisions which could affect pharmaceutical marketing rules. The TPP includes a provision focused on digital (online) marketing of pharmaceuticals to health professionals and consumers (Annex 26-A Art 4). This provision was incorporated into the CPTPP, and reproduced in almost identical form in the USMCA (Art 29.8).

At first glance this provision appears to require countries to allow the dissemination of information to health professionals and consumers via the internet. However, the first part of the provision (“As is permitted to be disseminated under the Party’s laws, regulations and procedures”) means that countries that currently prohibit or restrict these types of advertising can continue to do so [44]. In each of the agreements, the provision is not enforceable through state-to-state dispute settlement. The provision could, however, be perceived as affecting investor rights, potentially bolstering ISDS claims over attempts to prohibit or restrict direct-to-consumer advertising (DTCA) or marketing to health professionals [44]. Aside from these provisions directly targeting regulation of pharmaceutical marketing, all four agreements also feature Cross-Border Trade in Services chapters (CETA Chapter 9, TPP/CPTPP Chapter 10 and USMCA Chapter 15) with rules that may frustrate efforts to regulate pharmaceutical marketing, unless Parties explicitly exclude pharmaceutical advertising services from their coverage. Examples of provisions include rules that prohibit restrictions on market access, including bans and other quantitative restrictions (CETA Art. 9.6, TPP/CPTPP Art 10.5, USMCA Art. 15.5), and rules that prevent Parties from requiring that cross-border service suppliers have a local presence (TPP/CPTPP Art 10.6, USMCA Art 15.6), which can make regulations difficult to enforce.

The evidence base regarding DTCA of pharmaceuticals is still developing, but DTCA has been shown to stimulate demand for patented drugs (thereby increasing expenditure), and to interfere with rational prescribing [45]. Pharmaceutical promotion expenditure in the USA focuses on new drugs that are likely to generate significant returns on investment, and television advertisements are more concerned with promotion than education [46]. A recent systematic review of the effects on prescribing when physicians receive information directly from pharmaceutical companies found evidence of an association with a deterioration in prescribing appropriateness and a rise in prescribing costs and frequency [47].

### Regulatory requirements for assessment of safety, efficacy and quality

The TPP, CPTPP, and USMCA each include regulatory requirements for assessment of safety and efficacy, including marketing authorization and pharmaceutical inspections. The language and presentation of these provisions differ slightly between the TPP/CPTPP and USMCA, but the content is very similar.

Both agreements include articles directed towards harmonizing marketing authorization processes and aligning these with international and regional standards. Article 7 of TPP Annex 8-C and Art. 12.F.4 of the USMCA commit the Parties to improve the alignment of their regulations and regulatory activities through international initiatives, “such as those aimed at harmonization, as well as regional initiatives that support those international initiatives”. A further article (TPP Annex 8-C Art. 8 and USMCA Art. 12.F.6) requires countries to consider “relevant scientific or technical guidance documents developed through international collaborative efforts” and encourages them to “consider regionally-developed scientific or technical guidance documents” that are aligned with these international efforts. Furthermore, Article 16 of the TPP Annex 8-C and Article 12.F.6 para 10 of the USMCA require parties to review marketing authorization applications in a format consistent with the *International Conference on Harmonisation of Technical Requirements for Registration of Pharmaceuticals for Human Use: Common Technical Document*. Regulations for pharmaceutical inspections are also to be based on guidance documents developed through international collaborative efforts (TPP Annex 8-C Art. 18 and USMCA Art. 12.F.5 para 8).

Some forms of regulatory harmonization for pharmaceuticals can be advantageous for expediting registration of quality medicines and improving post-market surveillance and pharmaco-vigilance. It is unclear, however, whether the harmonization that is promoted by the provisions in the TPP/CPTPP and USMCA will result in improvements to the assessment of safety, efficacy, or quality—it may instead serve as a means to raise barriers in order to protect market shares and eliminate some of the competition. The primary forum through which harmonization in pharmaceutical regulation occurs is the International Council for Harmonisation of Technical Requirements for Pharmaceuticals for Human Use (ICH). Established in the early 1990s by industry associations and regulatory agencies in the USA, EU and Japan (headquarters to the bulk of the world’s multinational pharmaceutical companies), the main purpose of the ICH is to reduce the cost of developing pharmaceuticals, minimize regulatory requirements, and speed up marketing approval processes in order to promote market access [48]. It has been criticized for focusing on industry priorities such as promoting the economic interests of the large multinational pharmaceutical companies at the expense of smaller generic companies, developing countries, and patients [49]; for driving harmonization in a downwards direction towards the lowest common denominator [48]; and neglecting topics that would have clearer benefits for patient safety, such as the registration of clinical trials, patient information leaflets, and the release of information about adverse drug reactions [48].

CETA takes a somewhat different approach, including a *Protocol on the mutual recognition of the compliance and enforcement programme regarding good manufacturing practices for pharmaceutical products*, intended “to strengthen the cooperation between the authorities of the Parties in ensuring that medicinal products and drugs meet appropriate quality standards through the mutual recognition of certificates of GMP [Good Manufacturing Practice] compliance” (Art. 2). Depending on how this is done, mutual recognition and cooperation between regulatory agencies on inspection issues may result in improvements to consumer safety.

Both the TPP and USMCA stipulate the criteria that can be used to make marketing approval decisions with respect to the information required for demonstrating safety, efficacy, and quality (TPP Annex 8-C Art 11, also incorporated into the CPTPP; USMCA Art 12.F.6). Requiring sales and financial data is explicitly ruled out, and Parties are to “endeavor” not to require pricing data for making a marketing authorization decision. The absence of sales and financial data may not in itself present a problem, as these are not used in making marketing approval decisions in most countries—but if the list of criteria to be used is interpreted as a restrictive list, it may also rule out the use of other types of additional criteria, such as the “medical need” test used in Norway before it joined the European Medicines Agency [14]. Depending on the interpretation, these clauses might limit the levers available to prevent predatory pricing.

TPP Annex 8-C Article 12 obliges parties to administer marketing approval processes in a “timely, reasonable, objective, transparent, and impartial manner;” the USMCA includes a similar clause (Art. 12.F.6 para 4). However, pressure to speed up regulatory approval processes may compromise the safety of products entering the market [14]. With new drugs approved in Canada 1995–2010, the rate of serious safety issues was higher for drugs that had been given a priority (shorter) review than for those subjected to a standard review [50].

Both agreements also contain provisions (TPP Annex 8-C Art. 17 and USMCA Art. 12.F.5) regarding pharmaceutical inspections. Prior to conducting an inspection on another Party’s territory, Parties are to notify the other Party (“unless there are reasonable grounds to believe that doing so could prejudice the effectiveness of the inspection”), permit representatives of the other Party’s authority to observe the inspection (where “practicable”), and notify the other Party of its findings prior to public release, where this occurs. However, there is no requirement for public release of the findings of inspections. The USMCA contains additional clauses that promote the exchange of confidential information between the Parties. However, as Parties must prevent the disclosure of this confidential information, public release of information could be constrained when an inspection report is received from a country with more restrictive transparency standards. While CETA (Good Manufacturing Practices Protocol, Art. 14) and USMCA (Art. 12.F.5 para 6) restrict disclosure of confidential information only as it relates to good manufacturing practices, TPP and CPTPP (Art. 8.6.4) extend these restrictions to conformity assessment, which could also affect important safety data about pharmaceutical products.

A further provision, incorporated in TPP Chapter 8 and reproduced in USMCA Chapter 11, requires members to provide opportunities for “persons of another Party to participate in the development of technical regulations, standards and conformity assessment procedures by its central government bodies on terms no less favorable than those that it accords to its own persons” (TPP Art. 8.7.1; USMCA Art. 11.7.1). A footnote to each agreement allows Parties to limit this obligation to “providing interested persons a reasonable opportunity to provide comments on the measure it proposes to develop” and “taking those comments into account in the development of the measure.” The USMCA goes further here, with a provision requiring Parties to “allow persons of another Party” to participate in groups or committees that develop standards, on “no less favorable terms than its own persons” (Article 11.7.8). These provisions might enable pharmaceutical industry stakeholders to influence drug decision-making in other countries.

### Reduction/elimination of tariffs on pharmaceuticals or their ingredients

The TPP, CPTPP, and USMCA each eliminate tariffs on medicines for some countries and/or some pharmaceuticals. For example, Vietnam agreed to eliminate tariffs on medicines over a period of 10 years for CPTPP members [51] and Mexico’s tariff schedule for the USMCA eliminates tariffs on some medicines, including those based on rituximab and medicines containing erythropoietin [52].

Reducing or eliminating tariffs on medicines or their ingredients may contribute towards reducing the cost of medicines for consumers or payers, at least for imported medicines. This is by no means certain, however, as additional mark-ups at other points in the supply chain may elevate prices after the ingredient or product enters the market [53].

Some LMICs maintain tariffs on finished pharmaceutical products to protect their fledgling domestic generic manufacturers from foreign competition [54]. Local production of pharmaceuticals in LMICs may improve access to medicines by increasing price competition (thereby driving down prices); ensure that the country’s needs for specific generic medicines are met regardless of the priorities of pharmaceutical industries in other countries (e.g., India); and maximize efficiencies through relationships with distribution networks [55]. Local production can also enable redundant sources of supply to maintain a healthy market and protect against stockouts due to problems with production and supply [18]. Maintaining and building a viable domestic pharmaceutical industry also contributes to economic and development goals [55] and may be politically important even when not economically attractive in a static, immediate sense.

Most high-income countries have already eliminated tariffs on medicines, whether unilaterally or through reciprocal arrangements under the WTO GATT 1994 Communication on Trade in Pharmaceutical Products [54, 56], and tariffs on pharmaceuticals maintained by LMICs have been gradually falling [54]. A few countries (including India) continue to apply relatively high tariffs [53], however. The proportion of global pharmaceutical trade involving countries that continue to maintain tariffs increased between 2006 and 2013, meaning that a growing proportion of pharmaceutical trade is subject to tariffs [53]. In this context, newer trade agreements may still play a significant role in reducing tariffs on medicines.

While the elimination of pharmaceutical tariffs in the context of multilateral trade relations is generally deemed advantageous for access to medicines, the effects of preferential trade agreements are less clear, because of several complexities. Much depends on how the pharmaceutical market is structured domestically. Over-reliance on imports of pharmaceuticals and pharmaceutical inputs from certain markets may create vulnerabilities if there is instability in the market, as well as the potential for negative impacts on the domestic pharmaceutical sector [57].

The appropriate balance between importing pharmaceuticals and local production is country-specific: in some cases, importation may be more effective financially. However, in some contexts, tariffs may serve as important tools for navigating the tensions between the objectives of affordability and the benefits of local production. Agreeing to binding commitments in trade agreements may involve sacrificing some policy flexibility in making adjustments or trade-offs, in order to maximize benefits.

### Rules applying to government procurement of pharmaceuticals

The TPP, CETA, and USMCA each contain government procurement chapters. The purpose of these chapters is to ensure that governments or government entities purchasing goods and services (above certain monetary thresholds) do not discriminate against suppliers from another Party, or against local suppliers that are affiliated with or owned by foreign entities from another Party, or that provide goods and services from another Party.

The TPP Government Procurement Chapter (Ch. 15), retained almost entirely in the CPTPP [19],^13^ is based closely on the rules contained in the WTO Government Procurement Agreement (GPA). However, only a few TPP countries are currently members of the GPA,^14^ and procurement markets in Vietnam, Malaysia, and Brunei have been mostly closed to date [58]. However, these countries have long transition periods for implementing the CPTPP government procurement obligations.

The rules of TPP/CPTPP Chapter 15 apply, for the most part, only to those entities listed in a series of annexes. For most countries, the rules apply to all goods and services except those that are specifically excluded. All TPP parties appear to have made commitments to allow suppliers from other TPP parties to bid for pharmaceutical government procurement contracts at the national or subnational level, or both [59]. However, determining how much of a particular country’s pharmaceutical procurement is covered would require a detailed analysis of all institutions involved in purchasing pharmaceuticals in each country. Commitments have also been made on government purchasing by health ministries, including purchasing by public hospitals (Malaysia) and 34 state-owned hospitals (Vietnam) [59]. Vietnam’s liberalization of government procurement of pharmaceutical products is to be phased in gradually over 16 years, and is to cover only 50% of the contract value for pharmaceutical products at the end of that time [60]. Governments, however, also establish thresholds below which there is no obligation to liberalize a procurement contract. These specifications by different Parties make it difficult to draw general conclusions as to whether liberalized tendering, when the contract is large enough to cross the threshold, will assist in lowering drug costs (through competitive pricing) or will, over time, increase drug costs (by forcing initially less competitive suppliers out of the market and contributing to an eventual monopoly) [57].

The CETA Government Procurement Chapter (Ch.19) contains a similar set of rules. Canada and most member countries of the European Union have listed their departments or ministries of health and/or other agencies with responsibility for pharmaceuticals.

The USMCA Government Procurement Chapter (Ch. 13), which is very similar to the corresponding TPP chapter, applies only to the USA and Mexico. The US Department of Health and Human Services is covered, and pharmaceuticals are not excluded from the scope of covered goods; but sub-national entities that may be involved in procuring pharmaceuticals in the USA are not covered. Mexico has listed its *Comisión Federal para la Protección contra Riesgos Sanitarios* (Federal Commission for Protection against Health Risks), and pharmaceuticals are not excluded from the scope of coverage. Mexico has set the threshold for tendering quite low, although procurement contracts up to a certain value can be set aside for Mexican suppliers.

Exceptions for measures necessary to protect health are included in each government procurement chapter; however, the exceptions are subject to the requirement that measures are not to be applied in an arbitrary or discriminatory manner, or are not a disguised restriction on trade. Such exceptions may assist Parties in defending a measure in the case of a dispute, but this is by no means certain.

The effects of opening government procurement of pharmaceuticals through trade agreements have not been the subject of published research. Open, competitive tendering may result in lower prices for pharmaceuticals and is one of the strategies recommended by WHO [61]. However, tendering can, in the longer term, have negative impacts such as forcing some suppliers out of the market and reducing competition, potentially contributing to shortages [61]. Liberalizing government procurement may also affect the viability of fledgling generic medicines industries in those countries where local pharmaceutical companies are dependent, at least in the short term, on preferential arrangements.

### Rules applying to state-owned enterprises and designated monopolies (SOEs)

Under the relevant chapters on state-owned enterprises and designated monopolies (TPP/CPTPP Ch. 17, CETA Ch. 18, and USMCA Ch. 22), states retain the right to maintain and establish state-owned enterprises and monopolies, but these entities must operate according to certain rules to ensure that they do not have a competitive advantage over other firms. CETA defines a state enterprise as “an enterprise that is owned or controlled by a Party” (Ch. 1, Art. 1.1). State Owned Enterprises (SOEs) are defined in TPP Article 17.1 and USMCA Article 22.1 as enterprises “principally engaged in commercial activities”, in which the government owns more than 50% of the shares, exercises more than 50% of the voting rights or has the power to appoint the majority of the board or management body. This covers SOEs that have mixed commercial and other purposes, and applies regardless of whether they actually make a profit. The USMCA definition is broader, capturing enterprises where the government *indirectly* holds more than 50% of shares, or holds the power to control the enterprise through another ownership enterprise.

A core principle for each of these chapters is that state-owned enterprises and monopolies, when engaging in commercial activities, must act in accordance with commercial considerations (i.e., like a private business) in the purchase or sale of goods and services,^15^ and in ways that do not discriminate against the goods or services of another party [62]. These rules restrict preferential procurement from local producers by SOEs. TPP Chapter 17 represented a significant development in the scope and level of detail of provisions for SOEs in trade agreements [62]. The chapter’s legal rules were retained in their entirety in the CPTPP.^16^ The requirements of the TPP and USMCA are more extensive than those of CETA, preventing Parties from providing non-commercial assistance to state-owned enterprises where this would cause adverse effects to the interests of another Party. “Non-commercial assistance” refers to assistance provided as a result of state ownership or control, and includes financial assistance (e.g. transfers of funds, grants, debt forgiveness and loans) and other forms of favorable treatment, such as shared distribution networks or R&D support [62]. Article 22.6 of the USMCA goes even further than the TPP in prohibiting certain forms of non-commercial assistance altogether.

These provisions have potential implications for state-owned pharmaceutical companies in LMICs such as Vietnam. The Vietnamese domestic pharmaceutical industry is still in a nascent stage, with most raw materials imported, and involving (primarily small) local companies characterized by inefficient processes, low financial and R&D capacity, and outdated technology [63]. The Government of Vietnam has introduced a series of policies aimed at developing the country’s pharmaceutical industry to the point where it will be able to meet domestic demand [63]. If this is to be achieved, local firms, including SOEs, are likely to need subsidies and other types of support in order to become more competitive [63]. However, the CPTPP SOE rules limit the government’s possibilities for providing financial support and preferential treatment to develop these domestic firms.^17^ It is important to note, however, that pressure to reform inefficient SOEs may, under some circumstances, result in greater competition and lower prices.

### Procedural requirements for customs administration and trade facilitation

All four agreements include chapters concerning the administration of customs procedures, focused mainly on ensuring that customs regulations and procedures are transparent, predictable, and streamlined, and that they facilitate the movement of goods across borders. However, TPP Chapter 5 (Customs Administration and Trade Facilitation), and the corresponding USMCA Chapter 7, contain provisions regarding the exchange of information on customs issues—for example, TPP Article 5.2.3, which requires Parties to respond to written requests for information “If a Party has a reasonable suspicion of unlawful activity related to its laws or regulations governing importations.” Article 7.21 of the USMCA goes further, requiring cooperation between the Parties on border inspections, including the examination of goods. It is possible that, in the context of TRIPS-Plus enforcement of intellectual property rights, these provisions may frustrate the movement of generic pharmaceuticals across borders in cases where they are suspected of being counterfeit goods, i.e., goods suspected of violating IP rules rather than being of deliberately inferior quality. However, as these chapters are clearly intended to *facilitate* the cross-border movement of goods, it is also conceivable that cooperation between the Parties on customs issues and border inspections may make such seizures less likely, or may facilitate more rapid resolution.

### Rules applying to regulatory practices, cooperation and coherence

All four agreements include chapters dedicated to what is variously referred to as “regulatory cooperation” (CETA Ch. 21), “regulatory coherence” (TPP/CPTPP Ch. 25), and “good regulatory practices” (USMCA Ch. 28). CETA focuses on cooperation between the Parties through the creation of a Regulatory Cooperation Forum (Art. 21.6). The TPP/CPTPP agreements include provisions addressing how regulations are developed at the domestic level. The TPP Regulatory Coherence Chapter represented a significant normative development in terms of embedding these types of provisions in trade rules [64]; the USMCA has taken this further, with deeper, broader and more binding commitments.

The TPP/CPTPP Regulatory Coherence chapter encourages Parties to undertake regulatory impact assessments, following specific processes (Art 25.5). They are encouraged to assess the need for a regulatory proposal, examine feasible alternatives, explain the grounds for concluding that the approach selected will achieve the policy objectives, rely on the best available information, and provide easy-to-understand publicly accessible information. The TPP’s dispute settlement processes do not apply to this chapter, meaning that one Party cannot force another to comply.

In contrast, USMCA Chapter 28 contains a far more prescriptive and detailed set of requirements, with most provisions couched in binding legal language (each Party “shall”…), and is also enforceable through the USMCA’s dispute settlement process, at least for “a sustained or recurring course of action or inaction that is inconsistent with a provision of this Chapter” (Art 28.20). The main concern regarding pharmaceutical policy is that the detailed requirements for developing domestic regulatory measures may provide the industry with grounds for complaints (e.g., that the exploration of feasible alternatives, information used in decision-making, and/or information provided about the proposed regulatory measures were inadequate). Furthermore, where expert advisory groups are used for providing advice to regulatory authorities, each Party to the USMCA must “encourage its regulatory authorities to ensure that the membership of any expert group or body includes a range and diversity of views and interests, as appropriate to the particular context” (Art 28.10.3), and must endeavor to provide “means for interested persons to provide inputs to the expert groups or bodies” (Art 28.10.5). This may be used to justify industry membership of, or input to, expert groups and bodies, and serve to frustrate efforts to avoid conflicts of interest in the development of pharmaceutical policy.

Finally, all four agreements also include rules in other chapters (CETA Ch. 27, TPP/CPTPP Ch. 26, USMCA Ch. 29) that apply to the development of domestic regulations, such as those requiring prompt publication of proposed laws, regulations, procedures, and administrative rulings, and the provision of “reasonable opportunity” for “interested persons” and other Parties to comment on such proposals (CETA Art 21.1, TPP/CPTPP Art 26.2, USMCA Art 29.2). These provisions add further “red tape” and potential opportunities for industry influence in policy-making.

## Discussion and conclusion

The analysis presented here has indicated the substantial range of provisions and pathways in need of further exploration, beyond IP protection as such, with potential impacts on pharmaceutical policy that extend beyond the issues of access and affordability. Some of these provisions (such as regulatory requirements for assessing safety, efficacy, and quality; rules for SOEs and regulatory coherence) have appeared in trade agreements only recently and have been subjected to scant analysis and little or no empirical research, as they are only beginning to be adopted and implemented. The analytical framework proposed in Table 1 brings these provisions together into a comprehensive checklist of provisions, pathways, and potential impacts.

We envisage that the analytical framework may be useful as:

- a guide to the types of provisions and potential impacts that the health and human rights impact assessments of proposed trade and investment agreements need to consider, to fully explore the potential impacts on pharmaceutical policy;
- a checklist for trade negotiators (and their health advisors) who scrutinize proposed legal texts for potential issues in need of closer examination, or for issues that health experts and non-governmental organizations engaged in advocacy want to put in focus;
- a template for analysis for countries that are considering joining existing trade agreements, such as the CPTPP, to assist them to identify the implications of an existing set of legal rules for their own health and pharmaceutical systems; and
- an analytic tool for researchers engaged in tracing the impacts of specific agreements on pharmaceutical policy.

Empirical study of the effects of TRIPS-Plus IPRs on access to medicines is complicated by the long time-frames before most of these provisions begin to affect the length of exclusivity and play out in terms of higher expenditure and prices or reduced access to affordable medicines [11]. By contrast, the effects of many of the other provisions analyzed here, while also challenging to measure empirically, may be observable much earlier. Attention needs be given to developing methods and tools for exploring the impact of the full range of pharmaceutical-relevant provisions now being included in trade agreements.

It is important to note that the analytic framework presented here only identifies provisions and pathways that may have *potential* impacts, whether positive or negative. There is considerable variation in the provisions included in different agreements and the specific legal language employed, including “constructive ambiguities” that leave interpretation unclear [65]. The actual impacts will depend on a myriad of factors which are specific to the trade agreement in question, the context in specific countries, and how agreements are interpreted through domestic legislation and through dispute resolution. Further, states can mitigate the impact of provisions in future trade agreements through careful negotiation (e.g., through exclusions, exceptions, and transition periods for implementation), or offset the impacts through compensatory strategies (e.g., price controls for pharmaceuticals).

There is no doubt that future trade agreements will continue to present a wide range of potential intersections with pharmaceutical policy which negotiating countries will need to grapple with in the context of efforts to achieve SDG 3.8. However, the analysis presented here indicates that there is not a simple progression of deepening commitments from one trade agreement to the next. The suspension of certain IP provisions and the procedural rules for pharmaceutical reimbursement programs in the CPTPP could be seen as signaling a retreat from the more extreme positions sought by the USA. However, the re-emergence of even more extreme provisions in the USMCA clearly indicates that the USA, when it holds pivotal negotiating power, will continue to push for increasingly stringent IPR provisions.

A final point: the provisions discussed here may have impacts on the ability of countries to achieve SDG 3.8 by other pathways in addition to those that affect the four core pharmaceutical objectives. For example, to the extent that any of these provisions increase public costs with little or no improved therapeutic benefit, they become opportunity costs as regards achieving SDG 3.8. Many of the provisions impose a considerable administrative burden of compliance on LMICs, with human resource and infrastructure implications. As Walls and colleagues point out, “If states do not find ways to increase their administrative regulatory capacities in regard to the negotiation, implementation and on-going management of PTAs [preferential trade agreements], these PTAs will potentially drive greater health inequities” [66].

Limitations of our study include that the framework is based solely on the contents of four recently negotiated trade agreements. Other trade agreements recently negotiated or currently under negotiation may include variations on the provisions described here, or may contain new provisions not featured in previous agreements. What we have presented is an overview of the pathways and potential impacts for the purpose of developing the analytic framework, rather than a detailed health impact assessment of the likely effects of the trade agreements in specific contexts.

Here we have offered an analytic framework linking ten types of provisions in regional trade agreements with potential impacts on four core pharmaceutical policy objectives, via a range of pathways. It is our hope that this framework may prove useful for future health and human rights impact assessment and research into the implications of trade agreements for pharmaceutical policy and access to medicines.

## Supplementary information

**Additional file 1.** Types of provisions in recent regional trade agreements relevant to pharmaceuticals, and location of relevant provisions in chapters, annexes and side instruments. The file provides a breakdown of the chapters, annexes, and side instruments in recent regional trade agreements containing provisions that could impact on domestic pharmaceutical policy and regulation.

**Additional file 2.** Specific TRIPS-Plus intellectual property provisions in recent regional trade agreements relevant to pharmaceuticals. The file identifies the article/section numbers in recent regional trade agreements where there are specific TRIPS-Plus intellectual property provisions that are relevant to pharmaceuticals.

## Data Availability

The datasets used and/or analyzed during the current study are available from the corresponding author on reasonable request.

## Abbreviations

GDP: Gross domestic product
GSC: Global supply chain
GVC: Global value chain
ICTs: Information and communication technologies
IP: Intellectual property
IPRs: Intellectual property rights
ISDS: Investor State Dispute Settlement
MNC: Multinational corporation
NCDs: Non-communicable diseases
OECD: Organization for Economic Cooperation and Development
TNC: Transnational corporation
TRIPs: Agreement on Trade-related Intellectual Property Rights
UHC: Universal Health Coverage
WHO: World Health Organization
WTO: World Trade Organization

## Footnotes

1 The North American Free Trade Agreement (NAFTA) involving Canada, Mexico and the United States, which came into effect on 01 January 1994, also contributed to a new global standard for IP and became a model for subsequent US trade agreements.

2 Health impact assessment (HIA) and human rights impact assessment (HRIA) are two related but distinct approaches which can be used to assess the potential or actual impacts of trade agreements. Methods follow similar steps, but HIA identifies impacts on health, including distributional impacts, whereas HRIA explicitly references potential impacts to states’ legal obligations with respect to the Right to Health. [16]. Gleeson D, Forman L. Implications of trade and investment agreements for access to affordable medicines and the right to health. Canadian Yearbook of Human Rights, In Press.

3 It will come into force for the remaining five countries as their domestic approval processes are completed.

4 Side instruments from the TPP were not included in the analysis, except for those which have been incorporated into the CPTPP (as many of the original side instruments became defunct or were superseded and are no longer publicly available).

5 Although the New Zealand Ministry of Foreign Affairs and Trade is the official repository of the TPP and CPTPP documents, some CPTPP side instruments not relevant to New Zealand were not located there and were sourced from governmental websites of other CPTPP Parties.

6 Chapters that form part of the architecture of the agreement but which would not be expected to have an impact on pharmaceutical policy independently of other parts of the text were not included in the framework. These include dispute settlement chapters and exceptions chapters. Each agreement incorporates the Article XX(b) health exception from the General Agreement on Tariffs and Trade (GATT XX(b)) and applies it to the obligations in a limited subset of chapters. GATT XX(b) can be invoked by Parties who wish to defend a measure that would otherwise be in breach of the agreement as ‘necessary to protect human, animal or plant life or health’, as long as they can show that the measure ‘does not constitute a means of arbitrary or unjustifiable discrimination between countries where the same conditions prevail, or a disguised restriction on international trade’. The likelihood that a health measure will be determined by a dispute settlement panel not to be in breach of an agreement when the GATT XX(b) exception is invoked is difficult to predict and depends on the specifics of the measure and its application and context.

7 TRIPS-Plus provisions can also impact prices and access through other mechanisms such as restrictions on compulsory licensing and parallel importation, restrictions on patent opposition and revocation, limitations on exclusions from patentability, and supplementary protection certificates. While these types of provisions have appeared in other trade agreements (and some were originally proposed for the TPP), none of the four agreements studied include such provisions in the final text. Some trade agreements, including the TPP/CPTPP and USMCA, also expect countries to be members of the Patent Cooperation Treaty (PCT). PCT membership is likely to increase the volume of patent applications filed locally and, with increased workload for examiners, perhaps the grant of patents that might otherwise be rejected, increasing the likelihood that more drugs have single suppliers. As all members of the agreements reviewed in this article are already members of the PCT, this particular provision will not change the existing situation for them (though it may restrict options for subsequent leaders). More generally, analyses of IP provisions in trade agreements should consider wither PCT membership is required.

8 Based on our reading of the literature ([30]. Jung Y, Kwon S. The effects of intellectual property rights on access to medicines and catastrophic expenditure. International Journal of Health Services 2015;45:507–529; [31]. Jung Y, Kwon S. How does stronger protection of intellectual property rights affect national pharmaceutical expenditure? An analysis of OECD countries. International Journal of Health Services 2018;48:685–701; [32]. Park W. Intellectual property rights and international innovation. In: Mascus K (ed). Frontiers of economics and globalization. Elsevier Science Vol 1:289–327.), there is limited support for the thesis that stronger IP will lead to more pharmaceutical innovation or better health outcomes; therefore, we have not dealt with the possibility in our analysis.

9 The case was found in favour of Australia, based on jurisdictional grounds.

10 Philip Morris Asia was ordered to pay a proportion of Australia’s legal costs defending its legislation as well as 50% of Australia’s share of the arbitration fees, leaving the country to cover A$12 million.

11 While the provisions applying to pharmaceutical pricing and reimbursement programs in the TPP/CPTPP and USMCA are procedural in nature, it is clear that the pharmaceutical industry has (largely unsuccessfully to date) sought to undermine price controls and reference pricing (Lopert & Gleeson, 2013).

12 The procedural requirements in TPP Annex 26-A would also not have applied to Canada or Mexico.

13 Two provisions in the Government Procurement Chapter are suspended by CPTPP Annex Paragraph 6. These provisions concern labor rights and the timing of further negotiations on government procurement.

14 While nearly all WTO agreements are binding on all members, the GPA is an exception to the “single undertaking,” a plurilateral agreement within the WTO that countries may decide to join, or not.

15 There are exceptions for conduct pursuant to a public mandate, but these are complex and untested.

16 The CPTPP Annex, paragraph 11, suspends certain obligations in the Annex IV Schedule of Malaysia, but these changes are not relevant to pharmaceuticals.

17 Non-conforming measures included in TPP Annex IV Schedule of Viet Nam (https://www.mfat.govt.nz/assets/Trans-Pacific-Partnership/Annexes/Annex-IV.-Viet-Nam.pdf) allow Vietnam to continue to provide some specific types of financial assistance under some circumstances. Other TPP countries have also used Annex IV to limit their obligations with respect to SOEs and designated monopolies.
