# Supplementary material for: Analyzing the impact of trade and investment agreements on pharmaceutical policy: provisions, pathways and potential impacts
Source: Global Health. 2019 Nov 28;15(Suppl 1):78. doi: 10.1186/s12992-019-0518-2 (PMC6882307; doi:10.1186/s12992-019-0518-2)
Supplement: Supplementary file 1 — Additional file 1. Types of provisions in recent regional trade agreements relevant to pharmaceuticals, and location of relevant provisions in chapters, annexes and side instruments. The file provides a breakdown of the chapters, annexes, and side instruments in recent regional trade agreements containing provisions that could impact on domestic pharmaceutical policy and regulation. [file 12992_2019_518_MOESM1_ESM.pdf]

Supplementary File 1: Types of provisions in recent regional trade agreements relevant to pharmaceuticals, and location of relevant provisions in chapters, annexes and side instruments

**Chapter/provision**	**TPP**	**CPTPP (TPP-11)**	**CETA**	**USMCA**
Intellectual property chapter containing TRIPS-Plus IP provisions	Chapter 18: Intellectual Property	Chapter 18 (some provisions suspended – see Supplementary File 2 for details)<br><br>Bilateral side letters between some countries regarding pharmaceutical marketing, Article 18.47 and 18.53	Chapter 20: Intellectual Property	Chapter 20: Intellectual Property
Investment protection: Investor-state dispute settlement mechanism; investment chapter with IP covered in definition of investment	Chapter 9: Investment	Incorporates TPP Chapter 9 (slightly narrowed scope)<br><br>Bilateral side instruments between New Zealand and Australia, Brunei, Malaysia, Peru and Viet Nam	Chapter 8: Investment and Annexes 8-A to 8-F<br>(Chapter 8 suspended until/unless ratified by each EU member state. Following signing of CETA, the ISDS rules were also substantially revised, calling for creation of an 'Investment Court System'.)	Annex 14-D (only applies between Mexico and U.S., scaled back)
Procedural requirements for national pharmaceutical pricing and reimbursement programs	Annex 26-A: Transparency and Procedural Fairness for Pharmaceutical Products and Medical Devices	The specific procedural rules in Annex 26-A Art 3 were suspended by CPTPP Article 2[1] (other	N/A	Chapter 29: Publication and Administration, Section B: Transparency and Procedural Fairness for Pharmaceutical Products and

[1] Article 2 of the CPTPP suspends certain provisions of the TPP until they are reinstated by consensus among the Parties.

	(not enforceable by state-to-state dispute settlement)	provisions are retained)		Medical Devices (not enforceable)
Provisions with implications for regulation of pharmaceutical marketing	Annex 26-A, Transparency and Procedural Fairness for Pharmaceutical Products and Medical Devices, Art 4 (not enforceable by state-to-state dispute settlement)	Incorporates TPP Annex 26-A, Art 4 (not enforceable by state-to-state dispute settlement)	N/A	Chapter 29: Publication and Administration, Section B: Transparency and Procedural Fairness for Pharmaceutical Products and Medical Devices Art 29.8 (not enforceable)
	Chapter 10: Cross-Border Trade in Services	Chapter 10: Cross-Border Trade in Services	Chapter 9: Cross-Border Trade in Services	Chapter 15: Cross-Border Trade in Services
Regulatory requirements for assessing safety, efficacy and quality	Chapter 8: Technical Barriers to Trade and Annex 8-C: Pharmaceuticals	Incorporates TPP Chapter 8 and Annex 8-C	Chapter 4: Technical Barriers to Trade, and Protocol on the Mutual Recognition of the Compliance and Enforcement Programme Regarding Good Manufacturing Practices for Pharmaceutical Products (and associated Annexes 1-3)	Chapter 11: Technical Barriers to Trade, and Chapter 12: Sectoral Annexes - Annex 12-F: Pharmaceuticals
Reduction/elimination of tariffs for pharmaceuticals or their ingredients	Chapter 2: National Treatment and Market Access for Goods Annex 2-D: Tariff Commitments	Incorporates TPP Chapter 2 and Annex 2-D	N/A	Chapter 2: National Treatment and Market Access for Goods (and associated annexes)
Rules applying to government procurement (including pharmaceuticals)	Chapter 15: Government Procurement, and	Incorporates TPP Chapter 15 and Annex	Chapter 19: Government Procurement	Chapter 13: Government Procurement, and

	Annex 15-A: Government Procurement	15-A with minor changes	Annex 19-A Market Access Schedule of Canada<br>Annex 19-B Market Access Schedule of the European Union	Annex 13-A (Government procurement schedules for Mexico and the US)
Rules applying to state-owned enterprises including pharmaceutical companies	Chapter 17: State-owned Enterprises and Designated Monopolies<br>Annex IV – State-Owned Enterprises and Designated Monopolies Non-Conforming Measures	Incorporates TPP Chapter 17 and Annex IV	Chapter 18: State Enterprises, Monopolies, and Enterprises Granted Special Rights or Privileges	Chapter 22: State-owned Enterprises and associated annexes, and Annex IV: Non-Conforming Activities
Procedural requirements for customs administration and trade facilitation	Chapter 5: Customs Administration and Trade Facilitation	Incorporates TPP Chapter 5	Chapter 6: Customs and Trade Facilitation	Chapter 7: Customs Administration and Trade Facilitation
Rules applying to regulatory practices, cooperation and coherence	Chapter 25: Regulatory Coherence	Incorporates TPP Chapter 25	Chapter 21: Regulatory Cooperation	Chapter 28: Good Regulatory Practices
	Chapter 26: Transparency and Anti-Corruption	Incorporates TPP Chapter 26 (apart from TPP Annex 26-A, Art 3)	Chapter 27: Transparency	Chapter 29: Publication and Administration
