# Supplementary material for: Analyzing the impact of trade and investment agreements on pharmaceutical policy: provisions, pathways and potential impacts
Source: Global Health. 2019 Nov 28;15(Suppl 1):78. doi: 10.1186/s12992-019-0518-2 (PMC6882307; doi:10.1186/s12992-019-0518-2)
Supplement: Supplementary file 2 — Additional file 2. Specific TRIPS-Plus intellectual property provisions in recent regional trade agreements relevant to pharmaceuticals. The file identifies the article/section numbers in recent regional trade agreements where there are specific TRIPS-Plus intellectual property provisions that are relevant to pharmaceuticals. [file 12992_2019_518_MOESM2_ESM.pdf]

Supplementary File 2: Specific TRIPS-Plus intellectual property provisions in recent regional trade agreements relevant to pharmaceuticals

Chapter/provision	TPP	CPTPP (TPP-11)	CETA	USMCA
Intellectual property chapter containing TRIPS-Plus IP provisions	Chapter 18: Intellectual Property	Incorporates TPP Chapter 18 (some provisions suspended by Article 2)	Chapter 20: Intellectual Property	Chapter 20: Intellectual Property
Patents for new uses/methods/processes	Art 18.37	Suspended by CPTPP Article 2	N/A	Art 20.36 para 2
Patent term adjustments for delays in granting patents	Art 18.46	Suspended by CPTPP Article 2	Article 20.27 (2-5 years based on period from filing of patent application to date of marketing approval)	Art 20.44
Patent term adjustments for delays in marketing approval process	Art 18.48	Suspended by CPTPP Article 2		Art 20.46
Data and/or market protection for new pharmaceutical products	Art 18.50	Suspended by CPTPP Article 2	Art 20.29 (6 years data protection + 2 years additional market protection)	Art 20.48 para 1
Data and/or market protection – additional 3 years for new indications/ formulations/methods of administration or 5 years for combination products	Art 18.50	Suspended by CPTPP Article 2	N/A	Art 20.48 para 2 (not required for parties providing at least 8 years of protection)
Longer period of data and/or market protection for biologics	Art 18.51 – at least 8 years or at least 5 years + other measures	Suspended by CPTPP Article 2	N/A	Art 20.49 10 years effective market protection
Patent linkage	Art 18.53	Incorporates TPP Art 18.53	Not required, but where countries have linkage systems Art 20.28 provides for a new right for originator manufacturers to appeal decisions under the Notice of Compliance linkage regulations	Art 20.51

Trade secrets protection – including both civil and criminal enforcement	Art 18.78	Incorporates Art 18.78	N/A	Section I
TRIPS-Plus enforcement including border measures for suspected counterfeit goods	Section I, including Art 18.76 (special requirements related to border measures)	Section I, including Art 18.76 (special requirements related to border measures)	Article 20.43	Section J, including Art 20.84
